# Influence of *IL10* and *TGFB1* Promoter Polymorphisms on Serum Cytokine Levels in Development and Severity of RA

**DOI:** 10.3390/ijms231911955

**Published:** 2022-10-08

**Authors:** Georgi Vasilev, Mariana Ivanova, Iskren Stanilov, Lyuba Miteva, Spaska Stanilova, Irena Manolova

**Affiliations:** 1 Laboratory of Clinical Immunology, Department of Clinical Immunology, Medical Faculty, University Hospital “St. Ivan Rilski”, Medical University, Sofia 1431, Bulgaria; 2 Clinic of Rheumatology, Department of Rheumatology, Medical Faculty, University Hospital “St. Ivan Rilski”, Medical University, Sofia 1431, Bulgaria; 3 Department of Molecular Biology, Immunology and Medical Genetics, Medical Faculty, Trakia Iniversity, Stara Zagora 6000, Bulgaria

**Keywords:** rheumatoid arthritis, IL-10, TGF-β1, rs1800896, rs1800469

## Abstract

In our study, we focused on the role of the immunosuppressive cytokines TGF-β1 and IL-10 in RA and, in particular, the influence of the *IL10*-1082 A/G (rs1800896) and *TGFB1*-509C/T (rs1800469) promoter polymorphisms on their levels as a prerequisite for RA and disease activity clinical features. We found significantly higher IL-10 and lower TGF-β1 serum levels in women with RA than in controls. Patients who carried the -1082AA and AG genotypes had significantly higher levels of lnIL-10 compared to GG in contrast to healthy women carrying the same genotypes. The heterozygous -1082AG genotype was less frequent in RA cases (45.4%) than in healthy women (56.1%) and could be a protective factor for RA development (over-dominant model, OR = 0.66 95% CI 0.38–1.57). In addition, RA patients carrying the heterozygous -1082AG genotype were less likely to be anti-CCP positive than those carrying the homozygous AA/GG genotypes (37.1% vs. 62.9%; OR = 0.495. 95% CI 0.238–1.029, *p* = 0.058). There was no association between TGFB1 -509C/T SNP and susceptibility to RA and no relation between systemic TGF-β1 levels and rs1800469 genotypes. In conclusion, the IL10-1082 genotypes affect the serum levels of IL-10 in women with RA in a different way from that in healthy women and appear to play a role in the genetic predisposition and autoantibody production in the Bulgarian population.

## 1. Introduction

Rheumatoid arthritis (RA) is an inflammatory rheumatic disease affecting the synovial tissue and leading to progressive joint damage associated with chronic pain, impaired functional capacity, and impaired quality of life. A total of 33% of patients cannot work five years after RA onset. In addition, RA patients have reduced life expectancy by 5–10 years [1]. The advent of biological and target synthetic DMARDs has revolutionized the understanding of treatment and long-term prognosis in RA. Biologics target specific inflammatory molecules that play a crucial role in disease pathogenesis [2]. Their use gives promising results in the fight against this systemic autoimmune disease by reducing the number of painful and swollen joints, reducing morning stiffness, normalizing the serum values of acute phase indicators, increasing the functional capacity of patients, as well as delaying the appearance of radiological changes, improving the quality of life of the patient and the prognosis of the disease [2]. For this reason, profound comprehension of the cytokine network in health and its dysregulations in the context of RA is conceptually central to developing next-generation therapies. In this line of thought, we can conditionally dichotomize cytokines into immunosuppressive and proinflammatory groups. In relative terms, the principal immunosuppressive cytokines are TGF-β1, IL-10, and IL-35, opposing a much larger number of proinflammatory ones, such as TNF-α, IL-1, IL-6, IL-12p80, IL-17, IL-23 [3].

In our study, we were interested in the role of the immunosuppressive cytokines TGF-β1 and IL-10 in RA and, in particular, the influence of the genetic terrain on their levels as a prerequisite for RA and disease activity clinical features. The expression level of IL-10 and TGF-β1 depends on different polymorphisms located mainly in its promoter and 5′UTR regions. Usually, these regions are highly polymorphic and significantly impact transcription and translation. The IL-10 gene is located on chromosome 1 (1q31-1q32) and has many polymorphic sites [4,5]. One of the most studied single nucleotide polymorphisms (SNP) located at position -1082 A/G (rs1800896) has been identified to correlate with IL-10 expression. It has been found that the transcription factor Sp1 requires variant allele -1082G to bind and realize its effect on the rate of increased transcription of IL-10 mRNA and protein production [6]. Many studies provide strong evidence for the association of this SNP (-1082A/G) with many immune-mediated diseases, as well as rheumatoid arthritis, such as the association with autoantibody production in patients with rheumatoid arthritis [7,8]. The TGF-β1 gene on chromosome 19 (19q13.1–13.3) encodes a 25-kDa multifunctional homodimer protein. Among known polymorphisms in the regulatory sequences of the TGF-β1, a promoter polymorphism -509C/T is the most studied. The dependency between -509C/T polymorphism and the plasma concentration of both acid-activated latent and active TGF-β1 has been reported by Grainger et al. [9]. This SNP is located within a regulatory consensus nucleotide sequence named YY1 sequences, which respond to the selective binding of transcription factor AP1. When the -509C allele is present in the *TGFB1* promoter, it can down-regulate the transcription rate of *TGFB1* [10]. A new paper reports the association of this SNP with disease activity in RA and may predict activity in different RA patient subgroups [11].

In this regard, the current study aimed to investigate whether *IL10*-1082A/G (rs1800896) and *TGFB1* -509C/T (rs1800469) genetic polymorphisms are associated with RA development and serum cytokine levels in the female Bulgarian population. In addition, IL-10 and TGF-β1 serum levels concerning disease characteristics were also assessed. 

## 2. Results

### 2.1. Association of IL10-1082A/G (rs1800896) and TGFB1-509C/T (rs1800469) SNPs with Susceptibility to RA

Genotype and allele frequencies of rs1800896 and rs1800469 among female RA patients and healthy women are presented in Table 1. The genotype distribution of IL10-1082A/G and TGFB1-509C/T polymorphisms was in agreement with HWE among RA cases (chi-squared = 0.841, *p* = 0.841; chi-squared = 0.44, *p* = 0.878, respectively) and controls (chi-squared = 3.33, *p* = 0.1; chi-squared = 0.451; *p* = 0.33, respectively). 

No association between the rs1800896 polymorphism and RA risk in Bulgarian women was established under the allelic model (allele A vs. allele G; OR = 1.04), the co-dominant model (GG vs. AA; OR = 1.24), the dominant model (AG + GG vs AA; OR = 0.82) and the recessive model (GG vs. AA + AG; OR = 1.52). Nevertheless, the heterozygous genotype AG was less frequent in RA cases (45.4%) than in healthy women (56.1%). Our findings suggest that the AG genotype might decrease the RA risk in women (over-dominant model: AG vs. AA + GG; OR = 0.66; 95% CI 0.38–1.57, *p* = 0.15), whereas the GG genotype is a RA risk factor (recessive model: GG vs. AA + AG; OR = 1.52; 95% CI 0.72–3.21, *p* = 0.27).

Concerning genotype distribution and allele frequencies of the second SNP (rs1800469), no statistically significant differences were found between cases and controls under the same genetic models as in Table 1. The genotype distribution of rs1800469 was almost identical between groups (co-dominant model: chi-squared = 0.145; df = 2, *p* = 0.93. This study’s results confirm no association between TGFB1 -509C/T polymorphism genotypes and susceptibility to RA in the investigated population.

To evaluate the possible interaction of the genetic variants in these loci with RA susceptibility, a combined two-loci model was designed using two dichotomous variables for each cytokine gene polymorphism. We used the over-dominant model for IL10-1082A/G; and for the TGFB1-509C/T, we used the dominant model. The frequencies of six genotype combinations are presented in Table 2. The evaluation of combined IL10 and TGFB1 genotypes frequencies showed an increase in -1082AA + GG/-509CC (20.2% vs. 13.5%) and a reduction of -1082AG/-508CC (16.0% vs. 21.9%; OR = 0.489, 95% CI 0.217–1.101, *p* = 0.082) as well -1082AG/-508T heterozygous -509 CT+TT subjects (29.4% vs. 34.2%; OR = 0.578, 95% CI 0.28–1.193, *p* = 0.136) in RA patients compared to controls, respectively. The heterozygous -1082AG genotype could be a protective factor for RA development, regardless of which genotype of rs1800469 combines. In general, multiple regression models for RA observed no significant effect of the interaction between IL10 rs1800896 and TGFB1 rs1800469.

### 2.2. Serum Levels of IL-10 and TGF-β1 in Female RA Patients in Relation to Disease Status and IL10 (rs1800896) or TGFB1 (rs1800469) Gene Polymorphisms

The total (mean ± SD) IL-10 levels in RA patients were significantly higher than in controls (34.1 ± 52.3 pg/mL vs. 12.9 ± 32.8 pg/mL, respectively; *p* < 0.01). Conversely, in women with RA, the total (mean ± SD) TGF-beta1 levels were significantly lower than those in healthy women (10.0 ± 6.1 ng/mL vs. 22.8 ± 15.0, respectively; *p* < 0.000).

We assessed the possible influence of disease status and the genetic variants of IL10-1082A/G and TGFB1 -509C/T on cytokine production using generalized linear models with the bootstrap resampling procedure with 1000 samples; 119 female RA patients were included. Levels of TGF-β1 and IL-10 showed right-skewed distributions; hence, natural logs of the cytokine levels were used in the analysis as dependent variables (lnTGF-β1 and lnIL-10). To control for confounding factors, we had to examine which other variables had a significant effect on levels of TGF-β1 and IL-10. For this reason, MANOVA (multivariate analysis of variance) was conducted, and DAS28-CRP, age at onset, disease duration, and type of therapy for RA displayed a significant influence on the multivariate distribution of lnTGF-beta1 and lnIL-10 (Pillais’Trace *p* = 0.001). Two independent generalized linear models for IL10 and TGFB1 polymorphisms were estimated, including age, disease duration, DAS28-CRP, and medical treatment therapy as covariates (Table 3 and Table 4). Patients who carried -1082AA and AG genotypes had significant higher levels of lnIL-10 compared to GG (adjusted *p* values; *p* = 0.006 and *p* = 0.006, respectively). The size of the effect was moderate (partial eta^2^ > 0.06). Age, DAS28-CRP and disease duration also had a significant effect on lnIL-10, with a moderate effect size. Of note, patients on csDMARDs also had significantly higher levels of lnIL-10 (adjusted *p* = 0.047). Regarding TGFB1 gene variants, patients who carried the CT genotype had significantly higher levels in comparison to the TT genotype (adjusted *p* = 0.049). The other comparisons were not significant. However, the power of the analysis to correctly reject the alternative hypothesis was insufficient. 

The mean (±SD) serum levels of IL-10 and TGF-β1 in female RA patients and healthy women stratified by rs1800896 and rs1800469 genotypes are presented in Table 5. Serum levels of study cytokines were evaluated in 100 female RA patients and 100 age-matched healthy women. A total of 19 RA patients who received tocilizumab were excluded from the analysis. 

Significant differences in IL-10 levels stratified by three genotypes of rs1800896 were found in the RA group (*p* = 0.024) but not in the control group (*p* = 0.252) (Kruskal–Wallis test). In RA, significantly lower IL-10 levels were observed in patients who carried the -1082GG genotype (11.47 ± 32.7 pg/mL) compared to the -1082AA genotype (48.8 ± 60.3 pg/mL, *p* = 0.046) and heterozygous AG genotype (34.0 ± 50.8 pg/mL, *p* = 0.036) (Mann–Whitney U test) In healthy women, the -1082GG genotype was linked to a higher IL-10 production (23.7 ± 46.4 pg/mL), however, without reaching statistical significance. The inter-group comparisons showed that serum concentrations of IL-10 were elevated in female RA patients who carried the AA and AG genotypes of rs1800896 compared to healthy women with the same genotypes (*p* = 0.014 and *p* < 0.001, respectively). Concerning homozygous genotype GG, no significant difference in serum levels of IL-10 was detected between cases and controls (*p* = 0.919, Mann–Whitney U test).

Concerning TGF-β1 levels in relation to rs1800469 genotypes, differences between the -509 C/T genotypes were found in RA patients with borderline significance (*p* = 0.068, Kruskal–Wallis test). We observed higher serum levels in patients who carried the heterozygous CT genotype (10.8 ± 5.8 ng/mL) compared to the TT (8.1 ± 6.1 ng/mL, *p* = 0.039) and CC (10.6 ± 6.5 ng/mL, *p* = 0.085) genotypes (Mann–Whitney U test). Within control groups, no significant differences in the circulating TGF-β1 regarding the genotypes were found (*p* = 0.789, Kruskal–Wallis test).

### 2.3. Association of IL10 (rs1800896) and TGFB1 (rs1800469) SNPs with RA Disease Characteristics

Disease characteristics of female RA patients were analyzed concerning the genotype of IL10-1082A/G (rs1800896) and TGFB1-509C/T (rs1800469) SNPs. RA patients carrying the heterozygous -1082AG genotype were less likely to be anti-CCP positive than those carrying the homozygous AA/GG genotypes (37.1% vs. 62.9%; OR = 0.495. 95% CI 0.238–1.029, *p* = 0.058). Additionally, RA patients carrying the TGFB1-509 T allele in the genotype (CT + TT) demonstrated a trend for early RA development, as 72.4% of the patients with young-onset carry these genotypes (OR = 2.085. 95% CI 0.969–4.844, *p* = 0.058). There were no significant associations between rs1800896 and rs1800469 with age, disease duration, DAS28-CRP, HAQ-DI, CRP levels, and RF positivity. Data are presented in Table 6. 

## 3. Discussion

This study found significantly higher IL-10 and lower TGF-β1 serum levels in women with RA than in controls. In addition, significant differences in IL-10 levels stratified by three genotypes of rs1800896 were found in the RA group. Conversely, we observed no relation between systemic levels of TGF-β1 and rs1800469 genotypes.

IL-10 and TGF-β1 are widely accepted as immunoregulatory cytokines with a critical role in restoring immune homeostasis and are hence tightly involved in autoimmune pathologies. Several studies have reported increased IL-10 in both the serum and the synovial fluid of patients with RA demonstrating the involvement of IL-10 in the clinical manifestation of RA [12,13,14]. Our current study confirms the increased IL-10 levels in RA cases on a systemic level. We should note that we limited our study to women in accordance with the known prevalence of RA among women, more than 3 folds, previously reported sex differences in IL-10 production and its anti-inflammatory properties [15,16]. 

IL-10 is involved in RA immunopathogenesis through many biological functions, including its suppressive effect on the synthesis of proinflammatory cytokines, such as TNF-a, regulation of the Treg and Th1/Th17 balance, and the polarizing of macrophages to M2 phenotype [17] NLRP3 inflammasome activation [18]. Following the above, the results of our study’s performed independent generalized linear models showed a significant relationship between IL-10 serum levels with RA activity measured by DAS28-CRP and disease duration (Table 3). For many years, IL-10 was logically accepted as a promising RA therapy target. However, the complex nature of RA immunopathogenesis is influenced by several intrinsic and extrinsic factors, and the pleiotropic nature of IL-10 makes this aim difficult. The previous clinical trials showed a limited therapeutic effect of IL-10, but the studies continue these days in an attempt to offer a new IL-10-based strategy for RA therapy [19]. 

In addition to the significant relation of serum IL-10 with age, DAS28-CRP, and disease duration, our analyses also showed a significant relation with the genotypes of the promoter polymorphism rs1800896 in the *IL10* gene. In the present study, we found an under-representation of the IL-10-1082 AG genotype and an over-representation of the *IL10*-1082 GG genotype in Bulgarian women with RA who were linked to significantly lower IL-10 serum levels. We observed the highest IL-10 levels in RA women carriers of the AA genotype compared to the cases with the AG and GG genotypes. Conversely, among the healthy women, carriers of the GG genotype displayed the highest IL-10 levels (Table 5). These results seem contradictory to previous studies, including some of ours [20,21], where the GG genotype was reported as a higher IL-10 producer. Smelaya et al. (2016) comprehensively summarized the data from studies published from the period 1998–2014, focusing on the functional significance of the genetic polymorphisms in genes encoding pro- and anti-inflammatory cytokines and TLRs in the context of pneumonia [22]. At least 20 of the included studies investigated the *IL10*-1082A/G polymorphism and most associated the *IL10*-1082G allele or haplotype containing this allele with higher IL-10 production. However, the observed association was often detected after in vitro stimulation with lipopolysaccharides (LPS) that were specific for Gram-negative bacterial cell walls. Larsson et al. demonstrated that the A to G nucleotide exchange at position 1082 in the *IL10* gene resulted in the binding of the Sp1 transcription factor and increased promoter activity following lipopolysaccharide (LPS) stimulation of B cells [6]. However, the functional role of rs1800896 on *IL10* expression and IL-10 secretion could be different in the context of Gram-positive bacterial infection, anti-tumor immune response, or autoimmune disorders. We may hypothesize that in RA patients, in chronic inflammation provoked by different antigens, the regulation of *IL10* gene expression is under the control of other *cis* and *trans* regulatory factors, resulting in higher levels in carriers of the AA genotype. 

Our study established no significant association between the rs1800896 polymorphism and RA risk under any of the studied genetic models. Previously associative studies of rs1800896 on RA risk and severity were contradictory [13,23,24]. A meta-analysis by Jiang et al. (2012) also showed high heterogeneity in the European subgroup and concluded that rs1800896 is not associated with RA [25]. Some works showed an association of *IL10*-1082A/G SNP with autoantibody production in patients with rheumatoid arthritis [7,8,14]. Our results are in the same direction, as we observed that RA patients carrying the heterozygous -1082AG genotype were less likely to develop anti-CCP antibodies than those carrying the homozygous AA/GG genotypes.

Based on the above, we may accept that IL-10 and the rs1800896 polymorphism in the *IL10* gene are involved in RA immunopathogenesis, although the relationship of genotype-phenotype probably depends on additional intrinsic and extrinsic factors.

Concerning another studied cytokine, TGF-β1, our data showed significantly lower serum levels in RA women than in healthy controls independently of the rs1800469 genotypes. We also observed that this genetic variant was not associated with age, disease duration, DAS28-CRP, HAQ-DI, CRP levels, and RF positivity. A tendency for a higher *TGFB1* -509 T allele frequency among the RA cases with early disease onset (Table 6) was observed.

The dual role of TGF-β1 was widely explored in the context of cancers and autoimmune disorders [26,27,28]. This may explain the great variety and contradictory data between studies about the role of TGF-β1 in regulating the immune response. It is well known that the principal function of TGF-β1 is to suppress T-cell proliferation and to drive Treg differentiation. However, TGF-β1, in combination with IL-6, is required for Th17 differentiation. In addition, the biological effects of TGF-β1 depend on the activated signal pathways in the target cells [26]. Considering the bipolar nature of TGF-β1, we may assume that the lower TGF-β1 level in RA cases is involved in maintaining the Th17-mediated inflammation. However, it should be noted that TGF-β1, unlike many cytokines, is secreted in latent form and should be activated to exert its biological effects. In our study, the serum level of total TGF-β1 was measured. Additionally, the local TGF-β1 activities could be opposite to the systemic ones, demonstrated in previous studies in rodent models of RA [28].

Regarding the significance of the rs1800469 genetic variant for RA risk, our data showed no significant association with RA susceptibility or disease duration and severity. However, the presence *TGFB1*-509 T allele in the genotype (CT + TT) could be a risk factor for young-onset RA among female RA patients with an OR of 2.085. Our data generally agree with some studies [11,29] and are contradictory to others [30]. Iriyoda et al. (2022) reported that the -509 C/T polymorphism was not associated with RA susceptibility [11], and they observed a significant association with RA severity in a mixed population from Brazil. Similarly, Kim et al. (2004) demonstrated a significant effect of the -509 C/T polymorphism in the *TGFB1* gene on the progression of joint destruction in RA cases [30]. In addition, it should be noted that Kim et al. have reported gender-related differences in the distribution of *TGFB1*-509 C/T genotypes [29]. The observed discrepancies in reported studies could be explained by ethnicity, sex differences, or genotyping methods.

In summary, our current study demonstrated significantly lower serum levels of TGF-β1 and a lack of significant association of -509 C/T polymorphism in *TGFB1* with RA risk and severity.

Some limitations of our study should be noted. Although several confounding factors were taken into account, we cannot exclude others that have the potential to influence the cytokine serum level. Additionally, we did not examine the relationship between the category of body mass index (BMI) and cytokine levels since both patients and control groups were relatively homogeneous in terms of their body weight with no obsessed subjects. Moreover, no changes in many cytokines, including IL-10, have been observed in overweight healthy individuals compared with those with normal BMI [31]. In addition, during the data analysis, we noted that tocoliziumab therapy cases presented predominantly extreme values and were excluded. Another limitation of our study is that we focused our research on two functional genetic variants in promoter regions of the target genes. Both with the most pronouncing functional effect but other variants in the same genes or other functionally related genes might also influence IL-10 and TGF-β1 levels.

## 4. Materials and Methods

### 4.1. Study Subjects

One hundred nineteen patients with RA attending the Rheumatology Clinic of University Hospital “St. Iv. Rilski” in Sofia were included in this cross-sectional study and compared with 155 healthy subjects. Regarding the well-known prevalence of RA among women compared to men, our present case-control study included all patients, and healthy controls were women. Both patients and controls were Bulgarian Caucasians.

The inclusion criteria for RA patients were women meeting ACR/EULAR 2010 RA Classification Criteria [32] aged ≥ 18 years. Exclusion criteria were: a history of other inflammatory rheumatological or autoimmune disorders; malignancy; BMI ≥ 29.9; significant unstable or uncontrolled acute or chronic disease could confound the study’s results and/or current active infection.

The age range of participants was 18 to 79 years, with a mean (± SD) age of 45.2 ± 14.0 years. The mean (±SD) disease duration was 9.5 ± 8.4 years (range 1–40). A total of 81 (68%) of the patients were rheumatoid factor (RF-IgM) positive, and 62 (52.1%) were anti-CCP positive. Furthermore, 58 (49%) patients had disease onset under age 45 (young-onset RA).

Disease activity in RA patients was measured the by Disease Activity Score 28, calculated using C-reactive protein level (DAS28-CRP) [33] via an online calculator. Disease activity was interpreted as follows: inactive disease–low disease activity, DAS28-CRP < 2.1; moderate activity, DAS28-CRP >2.1 and <5.1; and high–very high disease activity, DAS28-CRP ≥ 5.1. A total of 15 patients (12.6%) had low disease activity, 71 (59.7%) had moderate, and 33 (27.7%) patients had high disease activity.

The Health Assessment Questionnaire Disability Index (HAQ-DI) (Fries et al., 1982) was applied to assess a patient’s functional ability level. Regarding drug therapy, 67 (56.3%) patients were being treated with conventional synthetic disease-modifying anti-rheumatic drugs (csDMARDs), 19 (16%) with the biological agent tocilizumab as monotherapy, and 33 (27.7%) were receiving long-term low dose prednisolone (10 mg/day) throughout the data collection. The demographic and clinical characteristics of RA patients are summarized in Table 7.

Randomly selected 155 healthy women living in the same regions of Bulgaria (university and hospital staff or recruited from a health checkup program during the study period) served as controls. The exclusion criteria were aforementioned.

The institutional ethics committee approved this study (University Hospital St. Iv. Rilski, decision number 6, 29 November 2016), and all subjects signed informed consent following the ethical guidelines of the Helsinki Declaration.

### 4.2. Blood Samples and DNA Extraction

Blood samples were collected from all participants in EDTA tubes and gel/clot activator vacutainer tubes. Serum samples were frozen in small aliquots at −70 °C until the ELISA analysis. Genomic DNA was extracted using a genomic blood DNA purification kit (NucleoSpin Blood L, Macherey-Nagel, Duren, Germany) and stored at −80 °C until use. The concentration of resulting DNA was measured spectrophotometrically at 260 nm using a NanoVuetm Spectrophotometer 9 (GE Healthcare, Buckinghamshire, UK). The ratio of absorptions at 260 nm vs. 280 nm was used to assess the purity of DNA samples.

Genetic and cytokine analyses were carried out in the Department of Molecular Biology, Immunology and Medical Genetics, Medical Faculty, Trakia University, Stara Zagora.

### 4.3. Genetic Analysis

Genotyping for the -1082A/G polymorphism in the *IL10* gene (rs1800896) was performed by an amplification refractory mutation system (ARMS)–PCR assay using a forward primer specific for G allele 5′-CCTATCCCTACTTCCCCC-3′, a forward primer specific for A allele 5′-CCTATCCCTACTTCCCCT-3′ and a reverse generic primer 5′AGCAACCACTCCTCGTCGCAAC-3′. The parameters of thes PCR protocol were the same as described by Miteva et al. [19].

Genotyping for the -509C/T polymorphism in the *TGFB1* promoter (rs1800469) was performed by a restriction fragment length polymorphisms (RFLP)-PCR assay after amplification of the 153bp fragment with the forwarding primer: 5′–CAG TAA ATG TAT GGG GTC GCA G-3′ and reverse primer: 5′–GGT GTC AGT GGG AGG AGG G–3′ as described previously by Stanilova et al. [34].

PCR amplification was performed in a GeneAmp PCR System 9700 (Applied Biosystems, Foster City, CA, USA) using primers and PCR reagents purchased from Metabion GmbH (Planegg, Germany) and Thermo Scientific (Waltham, MA, USA), respectively. The rs1800469-amplified products were digested using the restriction enzyme Eco81I (10 U/mL). The PCR amplification and restriction results were visualized using the Herolab gel documentation system and its analysis software E.A.S.Y. (Herolab GmbH Laborgeräte, Wiesloch, Germany). In each PCR run, a heterozygous control template was used to ensure accuracy. For quality control, 10% of randomly selected samples containing both cases and controls were analyzed a second time without any discrepancies.

### 4.4. Quantification of Serum IL-10 and TGF-β1 Concentrations

Serum IL-10 and TGF-beta1 levels were measured using enzyme-linked immunosorbent assay (ELISA) kits (RayBiotech, Inc, GA 30092 for IL-10; Affymetrix eBioscience, Vienna, Austria for TGF-BETA1) according to the manufacturer’s instructions. Before assay, the latent TGF-β1 contained in patients’ sera was activated to the immunoreactive form using acid activation and neutralization. A standard curve constructed with the kit’s standards was used to determine the cytokine concentration expressed in picograms per mL (pg/mL) for IL-10 and nanograms per mL (ng/mL) for TGF-BETA1. Serum samples of patients and controls were run in duplicate and analysed in the same analytic batch. The minimum detectable level of IL-10 was less than 1.0 pg/mL and 8.6 pg/mL for TGF-beta1.

### 4.5. Statistical Analysis

All data for this study were analyzed using RStudio (V) and SPSS version 28.0 for Windows (SPSS Inc., Chicago, IL, USA). The goodness of fit to Hardy–Weinberg equilibrium (HWE), calculating the expected frequencies of each genotype and comparing them with the observed values for patients and healthy controls, was performed using a chi-squared test. Binary logistic regression analysis was used to estimate odds ratios (OR), expressed with their 95% confidence intervals (95% CI) for disease susceptibility about study SNPs. Five inheritance models, including an allelic model with the wild-type (common) allele as the reference as well as dominant, recessive, co-dominant, and over-dominant genetic models, were used to evaluate the impact of individual alleles and genotypes on the disease prevalence. When appropriate, the comparison of mean cytokine concentrations between female RA patients and healthy women was made by Mann–Whitney U tests or Kruskal–Wallis test. MANOVA and general linear models were used to assess the relationship between the natural logarithm of circulating IL-10 and TGF-β1 levels and studied SNPs.

## 5. Conclusions

In conclusion, the IL10-1082 genotypes affect the serum levels of IL-10 in women with RA in a different way from that in healthy women and appear to play a role in the genetic predisposition and autoantibody production in the Bulgarian population. We suggest that IL10 rs1800896 influences RA development by regulating the production of IL-10.

## Figures and Tables

**Table 1:** Allele and genotype frequencies of *IL10-1082 and TGFB1-509C/T* (*SNPs*) in RA patients and controls.

Locus		RA n = 119 (%)	Controls n = 155 (%)	OR (95% CI) *	*p*-Value
***IL10-1082A/G (rs1800896)***					
	Genotype				
Co-dominant model	AA	40 (34.0)	45 (29.0)	Reference	
	AG	54 (45.0)	87 (56.0)	0.71 (0.38–1.35)	0.96
	GG	25 (21.0)	23 (15.0)	1.24 (0.53–2.87)	0.58
Dominant model	AA	40 (33.6)	45 (29.0)	Reference	
	AG + GG	79 (66.4)	110 (71.0)	0.82 (0.45–1.50)	0.52
Recessive model	AA + AG	94 (79.0)	132 (85.2)	Reference	
	GG	25 (21.0)	23 (14.8)	1.52 (0.72–3.21)	0.27
Over-dominant model	AA + GG	65 (54.6)	68 (43.9)	Reference	
	AG	54 (45.4)	87 (56.1)	0.66 (0.38–1.57)	0.15
Allelic model	Allele				
	A	134 (56.0)	177 (57.0)	Reference	
	G	104 (44.0)	133 (43.0)	1.04 (0.69–1.57)	0.86
***TGFB1-509C/T (rs1800469)***					
	Genotype				
Co-dominant model	CC	43 (36.1)	55 (35.5)	Reference	
	CT	61 (51.3)	78 (50.3)	0.98 (0.53–1.82)	0.99
	TT	15 (12.6)	22 (14.2)	0.76 (0.31–1.85)	0.727
Dominant model	CC	43 (36.1)	55 (35.5)	Reference	
	CT + TT	76 (63.9)	100 (64.5)	0.93 (0.52–1.67)	0.81
Recessive model	CC + CT	104 (87.4)	133 (85.8)	Reference	
	TT	15 (12.6)	22 (14.2)	0.77 (0.34–174)	0.52
Over-dominant model	CC + TT	58 (48.7)	77 (49.7)	Reference	
	CT	61 (51.3)	78 (50.3)	1.06 (0.61–1.87)	0.83
Allelic model	Allele				
	C	147 (62.0)	188 (61)	Reference	
	T	91 (38.0)	122 (39)	0.90 (0.59–1.37)	0.61

* RA, rheumatoid arthritis; OR, odds ratio; CI, confidence interval. * Adjusted ORs and *p*-values for age present.

**Table 2:** Combined genotype distribution. Genotype frequencies of *IL10*-1082 (rs1800896) and *TGFB1*-509C/T (rs1800469) SNPs genotypes in RA patients and controls.

*IL10*-1082A/G (rs1800896)	*TGFB1*-509C/T (rs1800469)	RA	Controls	OR (CI 95 %)	*p*-Value *
AA + GG	CC	24 (20.2%)	21 (13.5%)	Reference	
AA + GG	CT + TT	41 (34.5%)	47 (30.3%)	0.763 (0.372–1.568)	0.462
AG	CC	19 (16%)	34 (21.9%)	0.489 (0.217–1.101)	0.082
AG	CT + TT	35 (29.4%)	53 (34.2%)	0.578 (0.28–1.193)	0.136

* RA, rheumatoid arthritis; OR, odds ratio; CI, confidence interval.

**Table 3:** Multivariate relationship between study gene polymorphism of *IL10* (rs1800896), age, disease duration, therapy for RA, and disease activity score (DAS28-CRP) with IL-10 serum levels, separately, established by a generalized linear model of log-transformed levels of IL-10 with bootstrap resampling (1000 samples); 119 female RA patients were included in the model.

Independent Variable	Dependent Variable: ln IL-10 (pg/mL)			
	Exponent of Parameter Estimate (SE)	*p*-Value	Partial Eta^2^	Power
*IL10-1082 A/G*				
AA	3.6 (1.59)	0.006	0.079	0.112
AG	3.5 (1.56)	0.006	0.078	0.513
GG	Reference			
Age (years)	1.02 (1.01)	0.045	0.043	0.522
Disease duration (years)	1.07 (1.02)	0.003	0.095	0.866
DAS28-CRP	1.49 (1.13)	0.002	0.095	0.800
Therapy				
Cortiosteroids	1.46 (1.68)	0.467	0.006	0.8
csDMARDs	2.68 (1.63)	0.047	0.042	0.8
Tocilizumab	Reference			

csDMARDs, conventional synthetic disease-modifying antirheumatic drugs; DAS28-CRP, Disease Activity Score 28 calculated using C-reactive protein level.

**Table 4:** Multivariate relationship between study gene polymorphism of *TGFB1* (rs1800469), age, disease duration, therapy for RA, and disease activity score (DAS28-CRP) with IL-10 serum levels, separately, established by a generalized linear model of log-transformed levels of IL-10 with bootstrap resampling (1000 samples); 119 female RA patients were included in the model.

Independent Variable	Dependent Variable: ln TGF-Beta1 ng/mL			
	Exponent of Parameter Estimate (SE)	*p*-Value	Partial Eta^2^	Power
*TGFB1-509C/T*				
CC	1.2 (1.16)	0.20	0.017	0.24
CT	1.33 (1.16)	0.049	0.038	0.47
TT	Reference			
Age (years)	1 (1.01)	0.79	0	0.05
Disease duration (years)	1 (1.01)	0.91	0	0.05
DAS28-CRP	1.03 (1.03)	0.393	0	0.14
Therapy				
Corticosteroids	1 (1.14)	0.91	0	0.05
csDMARDs,	1 (1.15)	0.79	0	0.05
Tocilizumab	Reference			

csDMARDs, conventional synthetic disease-modifying antirheumatic drugs; DAS28-CRP, Disease Activity Score 28 calculated using C-reactive protein level.

**Table 5:** Serum levels of *IL10* and *TGF-β1* in female RA patients and healthy women in relation to *IL10* and *TGFB1* gene polymorphisms, respectively.

	RA (n = 100)	Controls (n = 100)	
	Mean ± SD	Mean ± SD	*p*-Value *
Total IL-10 (pg/mL)	34.1 ± 52.3	12.9 ± 32.8	**<0.001**
rs1800896 genotypes			
AA	48.8 ± 60.3	7.0 ± 5.0	**0.014**
AG	34.0 ± 50.8	12.3 ± 35.0	**<0.001**
GG	11.47 ± 32.7	23.7 ± 46.4	**ns**
*p*-Value within the group** 0.024 ns * AA vs. GG *p* = 0.045; AG vs. GG *p* = 0.036 * AA vs. GG *p* = 0.55; AG vs. GG *p* = 0.156			
Total TGF-β1 (ng/mL)	10.0 ± 6.1	22.8 ± 15.0	<0.001
rs1800469 genotypes			
CC	9.6 ± 6.5	24.6 ± 17.0	<0.001
CT	10.8 ± 5.8	21.5 ± 12.6	<0.001
TT	8.1 ± 6.1	22.5 ± 16.0	<0.001
*p*-Value within the group ** 0.068 ns * CC vs. TT *p* = 0.085; CT vs. TT *p* = 0.039			

Data from RA patients with biological treatment were not included in the analysis. * Mann–Whitney U test, ** Kruskal–Wallis test, RA, rheumatoid arthritis.

**Table 6:** Disease characteristics of RA in relation to IL10 (rs1800896) and TGFB1 (rs1800469) polymorphisms. Data are presented as mean ± SD or percentage of positivity. Comparisons between groups were made by the Mann-Whitney U test or chi-squared test, as appropriate.

	rs1800896			rs1800469		
Parameter	AA + GG	AG	*p*-Value	CC	CT + TT	*p*-Value
Age (years)	44.6 ± 14.9	45.9 ± 14.1	0.818	47.0 + 13.3	44.2 + 14.3	0.197
Disease onset young-onset late-onset	51.7% 57.4%	48.2% 42.6%	0.536 T allele	27.6% 44.3% OR = 2.085	72.4% 55.7% 95% CI	0.058 0.969–4.844
Disease duration (years)	10.3 + 8.9	8.6 + 7.7	0.281	8.2 + 7.4	10.3 + 8.9	0.248
CRP	22.9 + 45.3	27.1 + 48.3	0.523	20.3 + 31.4	27.5 + 53.6	0.952
DAS28-CRP	4.6 + 1.2	4.8 + 1.4	0.468	4.6 + 1.4	4.7 + 1.3	0545
Disease activity						
<5.1 DAS28	56.0%	44.0%	0.652	35.7%	64.3%	0.882
≥5.1 DAS28	51.5%	48.6%		37.1%	62.9%	
HAQ-DI	1.4 + 0.7	1.3 + 0.7	0.341	1.2 + 0.7	1.4 + 0.8	0.357
RF status negative positive	51.65 56.1%	48.4 43.9.7%	0.682	38.7% 33.3%	61.3% 66.7%	0.605
CCP status negative positive AG genotype	45.6% 62.9% OR = 0.495	54.4% 37.1% 95% CI	0.058 0.238–1.029	31.6% 40.3%	68.4% 59.7%	0.321

CRP, C-reactive protein; DAS28-CRP, Disease Activity Score 28 calculated using C-reactive protein level; CCP, cyclic citrullinated peptide antibody; HAQ-DI, Health Assessment Questionnaire Disability Index; RA, rheumatoid arthritis; RF, rheumatoid factor; SD, standard deviation.

**Table 7:** Demographic and clinical characteristics of study population of RA patients.

	Female RA Patients	Healthy Women	*p*-Value
*n*	119	155	
Age (years)	45.2 ± 14.0	42.22 ± 10.0	0.05
Disease duration (years)	9.5 ± 8.4		
Patients with + RF (%)	(81) 68%		
Patients with + anti-CCP (%)	(62) 52.1%		
CRP (mg/L)	24.8 ± 46.5		
DAS28-CRP	4.7 ± 1.32		
HAQ-DI	1.35 ± 0.73		
Therapy			
Symptomatic (%)	33 (27.7)		
csDMARDs (%)	67 (56.3)		
Tocilizumab (%)	19 (16)		

Data are presented as mean ± SD. Anti-CCP, anti-cyclic citrullinated peptide antibody; csDMARDs, conventional synthetic DMARD; CRP, C-reactive protein; DAS28-CRP, Disease Activity Score 28 calculated using C-reactive protein level; HAQ-DI, Health Assessment Questionnaire Disability Index; RA, rheumatoid arthritis; RF, rheumatoid factor; SD, standard deviation.

## Data Availability

The data presented in this study are available upon request from the corresponding author. The data are not publicly available due to local regulations and hospital restrictions.
